# Optimizing Safe Dental Practice During the COVID-19 Pandemic: Recommendations Based on a Guide Developed for Dental Practices in China

**DOI:** 10.3389/fmed.2021.619357

**Published:** 2021-05-26

**Authors:** Li Li, Mianyan Zeng, Xiao Chen, Shuman Cai, Cuixia Xu, Wei Xia, Lijun Jiang, Xiaoyan Zou, Pei Chen, Mingdeng Rong

**Affiliations:** Department of Periodontology and Oral Implantology, Stomatological Hospital, Southern Medical University, Guangzhou, China

**Keywords:** COVID-19, SARS-CoV-2, infection control, dental clinics, public health, prevention, disinfection

## Abstract

The current global coronavirus disease 2019 (COVID-19) outbreak is still exerting severe global implications, and its development in various regions is complex and variable. The high risk of cross-infection poses a great challenge to the dental practice environment; it is therefore urgent to develop a set of pandemic prevention measures to ensure dental practice safety during the COVID-19 outbreak. Therefore, we combined the epidemiological characteristics of severe acute respiratory syndrome coronavirus 2 (SARS-CoV-2), public emergency measures for COVID-19, characteristics of dental practice, and relevant literature reports to develop a set of dynamic practice measures for dental practices in high-, medium-, and low-risk areas affected by COVID-19. This will help dental practices to achieve standard prevention and ensure their safe and smooth operation during the pandemic. It is hoped that these measures will provide a reference basis for dental hospitals and dental clinics in their care and pandemic prevention work.

## Introduction

From the beginning of 2019 to March 25, 2021, the cumulative number of confirmed cases of coronavirus disease 2019 (COVID-19) in the world is more than 125,700,000 and causing more than 2,756,000 deaths, which shows that COVID-19 is still spreading around the world. COVID-19 vaccines have been developed currently. However, due to the emergence of a variety of mutated strains of severe acute respiratory syndrome coronavirus 2 (SARS-CoV-2) (1), coupled with the uncertainty of vaccine efficacy, the pandemic is still severe and undoubtedly poses a huge threat and harm to the health of people worldwide. It also presents a great challenge to dental practices, which are characterized by a great demand for consultation and emergency treatment, as well as an environment that has a high level of spattering (2). This is greatly increasing the risk of cross-infection in the dental clinical practice process. Thus, how can dental staff establish effective protection to ensure safe and smooth dental practice implementation?

Studies have shown that the main routes of transmission of SARS-CoV-2 are respiratory droplets and contact transmission, with an additional possibility of air and aerosol transmission (prolonged, closed, high concentrations). Furthermore, SARS-CoV-2 can be isolated from stool, urine, and conjunctival secretions. Thus, attention should also be paid to contact or aerosol transmission caused by its contamination of the environment (3–6). It is well-known that instruments and operations commonly used in dentistry are prone to generate large amounts of droplets and aerosols, and dental care personnel are therefore at risk of inhaling large amounts of aerosols. SARS-CoV-2 can persist in aerosols for up to 3 h (7). Due to the direct contact operation on the dental cavity, the dental mucosa is considered a potential high-risk route for SARS-CoV-2 infection (8). Moreover, the complex structure of the turbine handpiece, saliva ejector, and dental chair drainage pipe that are not easily cleaned and disinfected and the use of sharp instruments during practice makes dental care personnel prone to occupational exposure (9). This may cause an increase in cross-infection among dentists, nurses, and patients and could become a difficult point for prevention and control during the COVID-19 pandemic. To truly and comprehensively identify studies on the prevention and control of the pandemic in dental practice, the authors used keywords such as “COVID-19,” “SARS-CoV-2,” “COVID-19 dental,” “dental,” and “infection control” on the PubMed and China National Knowledge Infrastructure (CNKI) websites. It was hoped that the literature would provide insights into the measures and experiences of dental practices in other countries during the COVID-19 pandemic (10–16). Among them, Italy, Latin America, and Poland recommend that patients should undergo pretesting and triage, as well as reinforce personal protective equipment (PPE) usage and hand hygiene. A mouthwash that is effective against COVID-19, disinfection and sterilization, rubber dams in dental practice should also be used. Singapore suggested a “Proposed color-coded framework for dental practice during the COVID-19 pandemic” (17). However, the measures described in these articles are still not detailed or specific enough and lack dynamic guidelines for pandemic prevention.

Therefore, there is an urgent need to develop scientific and comprehensive dynamic guidelines applicable to the dental clinic and dental hospital practice. This should be based on the characteristics and policies related to the COVID-19 pandemic in different periods and regions. It would help control infection sources in time, cut off the transmission pathways, protect the safety of the three parties involved (namely, doctors, nurses, and patients), and ensure the safe and smooth operationalization of dental practice work. Therefore, based on the development of the COVID-19 pandemic and the dental clinical practice, we summarized the practice measures implemented by Chinese dental clinics during the COVID-19 pandemic. The effectiveness of these dynamic measures has been confirmed because no dentists have been infected with COVID-19, except for a few dentists in Wuhan, who were reported to be infected with COVID-19 at the beginning of the outbreak, so we hope that these measures can guide domestic and foreign dental hospitals and dental clinics for smooth and safe practice during the COVID-19 pandemic. The specific practice measures are as follows.

## Be Guided by the COVID-19 Prevention Policies and Measures Issued by National and Regional Health Authorities

China issued eight editions of the Diagnosis and Treatment Protocol for COVID-19 from January 15, 2020, to August 19, 2020 (18). These were issued in conjunction with daily updates for areas at high, medium, and low risk of an outbreak, making important dynamic guidelines for people to recognize and implement effective measures against the COVID-19 outbreak. As of March 1, 2021, 48 online learning sessions on infection control during hospital outbreaks have been held (19). Meanwhile, Guangdong Province issued daily dynamic guidelines for hospital outbreak prevention and control according to the pandemic changes. All regional dental hospitals and dental clinics have adjusted their systems and measures for conducting the dental practice promptly according to the policies and measures issued by the state and local provinces and municipalities.

## Introduction of “Health Codes” and Communication Big Data Trip Cards for Rapid Identification of Patient Risk Levels

The “health code” is filled in by the individual using information such as the identity card number, location information, health status, exposure history, and travel and residence history. The platform reviews the information and grants the applicant a QR code in red, yellow, or green. Holders of red and yellow codes are subject to a 14- and 7-day intensive or home quarantine, respectively, while holders of green codes are allowed normal passage. This system dynamically assesses and regrants codes according to the risk level of the pandemic.

The Communication Big Data Trip Card is a software developed by the China Academy of Information and Communication Technology, in conjunction with three basic telecommunication companies, allowing mobile phone users to check information on all the cities and regions they visited in the past 14 days (20, 21).

These two “electronic bodies” can help dental institutions quickly identify the risk level of patients, reduce triage time, achieve the purpose of less exposure for people, and more proper government supervision, making a significant contribution to China in the control of pandemics (22).

However, there will be no digital identity for those who buy smartphones unconditionally or have behavioral and cognitive limitations. The operation of the health code considers the plight of “"people without codes” in advance, and coresidents can help people without codes apply for health codes. These can be saved or even printed and have a validity period of 14 days (23). Nonetheless, it is still difficult to implement these digital technologies in countries where smartphones and the Internet are not widely available.

Because of the large amount of personal data collected to apply for electronic identity, there is the possibility of privacy breaches, as some privacy advocates still refuse to apply for electronic identity. For now, without electronic identity, their daily travel will be impacted. In this regard, several recent studies have discussed the future of electronic identity after the pandemic (24–26). A number of issues have been highlighted, such as whether to upgrade, improve, or delete the electronic identity after the pandemic, whether the private information can be completely deleted, and whether it could be used by criminals. These are issues that China needs to resolve in the near future. The author believes that to prevent unauthorized access to such data that could be used for criminal activity, it is necessary to develop new privacy software and establish more ideal confidentiality systems and laws.

## Dental Institutions Should Improve All Pandemic Prevention Work and Policies Before Starting a Dental Practice

### Establish a COVID-19 Pandemic Prevention and Control Team to Strengthen Supervision and Guidance Work and Strive to Promote and Improve the Level of Nosocomial Infection Control

Through networking, train whole staff in all protocols, including the receiving process and in the use of protective equipment. All personnel must be trained and qualified before taking up their duties, and it is particularly important to strengthen training on infection control for the dental support staff; even if they are not involved in direct patient care, they are often in contact with the patients' environment, leading to the possibility of contact transmission at any time (27, 28).

### Implement Whole-Staff Health Confirmation Before Starting a Dental Practice

Hospital staff returning from low-risk areas will be required to return to work with a green code for health and with no abnormalities in their communication big data trip. Hospital staff returning from medium- and high-risk areas need to be isolated at home or centrally for 14 days and test negative for viral nucleic acid before returning to work. Daily temperature checks are required for all commuting to and from work, and no gatherings are allowed to avoid cluster infections. Regular nucleic acid testing for hospital staff should be carried out after starting dental practice to rule out nosocomial infections.

### Implementation of Online Booking and Dental Care Consultation Services

Online appointments should be implemented in separate time slots to control the number of people attending each time slot, and only one patient should be booked per hour to avoid crowding. Fill in the epidemiological questionnaire form during online appointments to initially screen high-risk individuals. Online appointment numbers should be limited to patients with acute conditions such as severe dental pain, pericoronitis, post-operative osteitis, dry socket or abscess, cellulitis, tooth fracture or dislocation, and certain emergency restorative procedures (29). Furthermore, non-urgent patients should be encouraged to postpone their visits. Simultaneously, an “Internet + telephone” consultation service should be developed to actively provide health assessment, consultation guidance, psychological guidance, and other services. Additionally, detailed epidemiological history should be collected to correctly guide patients to the dental clinic in an orderly manner to reduce the risk of infection (30).

### Development of Contingency Plans for COVID-19 Outbreaks in Dental Specialties

**Figure 1:**
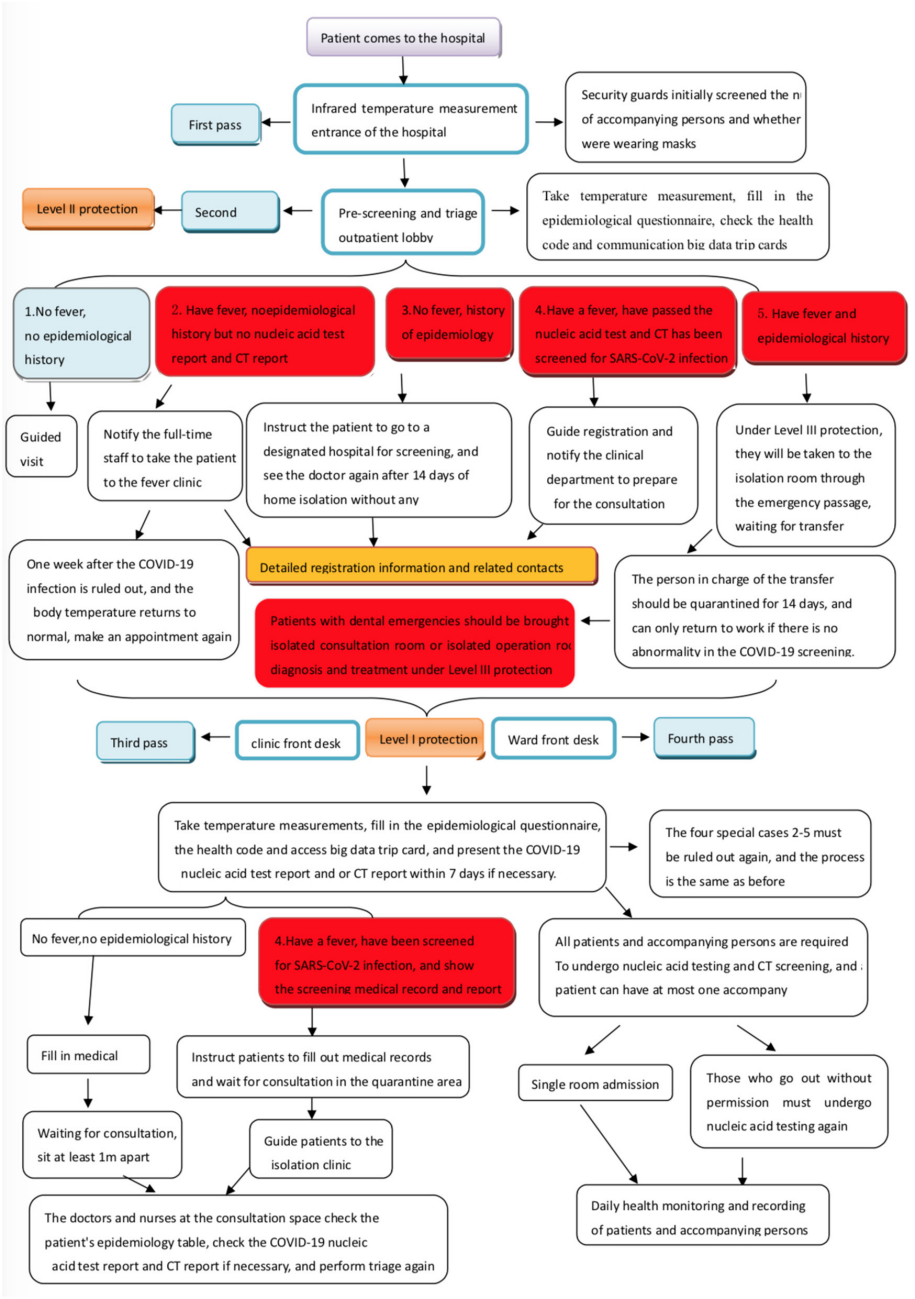
Pre-screening and triage process in dental institutions during the coronavirus disease 2019 (COVID-19) pandemic.

Since dental clinics are generally not designated clinics for treating COVID-19 patients, when four categories of people (confirmed cases, suspected cases, patients with febrile symptoms, and close contacts) are screened out, they need to be transferred to the designated COVID-19 clinics for further treatment (Figure 1). A separate isolation room should be set up at the location nearest to the dental practice facility's exit for temporary housing of patients pending transfer. The transfer route should be delineated to achieve the shortest outdoor distance and least contact with people (31), with daily access closed and only passable when the emergency plan is activated. Simultaneously, each department should set up a separate, well-ventilated, and disinfected single room as an isolation clinic and an isolation surgery room in the operating room, which is not open daily and is used only for the emergency treatment of these four categories of people. Because it is difficult to accurately predict the changes and development of the pandemic, it is important to reflect on the emergency management ability of hospitals to respond well to these changes (32).

Dental patients with fever or severe respiratory symptoms but no abnormalities in green health code or communication big data trip cards should be immediately notified to the department and the person in charge of COVID-19 infection control. Under secondary protection (see Table 1 for details), the receiver should lead the patient to the isolation room through a special emergency channel. The hospital's COVID-19 infection control staff should bring the patient to the fever clinic for consultation. After the nucleic acid test and computed tomography (CT) examination are found to be unremarkable, and a specialist rules out the COVID-19 infection, the patient can visit the dental clinic. After that patient leaves, the area through which he or she passes should be fully disinfected. The receiving staff should be isolated at home or centrally while awaiting the patient's screening results. If the results are unremarkable, the receiving staff can go to work normally; if the results are abnormal, they need to be observed in centralized isolation for 14 days. A negative nucleic acid test should be received before they can return to work.

**Table 1:** Requirements and scope of application of three levels of wearable protective.

**Protection level**	**Wearing requirements**	**Scope of application**
Level I	Disposable work caps, overalls, medical-surgical masks	The front desk of all outpatient departments and wards
Level II	Disposable work caps, overalls, medical-surgical masks or medical protective masks, goggles or protective face shields, disposable isolation suits, disposable latex gloves	Pre-screening triage staff in the primary outpatient lobby Tertiary clinic operators; exposure to patients with severe fever or respiratory symptoms but without unusual epidemiological history Cleaning personnel
Level III	Disposable work caps, overalls, medical-surgical masks or medical protective masks, goggles or protective face shields, protective clothing, disposable latex gloves, waterproof boot covers	Exposure to suspected or confirmed COVID-19 cases

If a dental patient is found to be in close contact of a suspected or confirmed COVID-19 case, the department and the person in charge of COVID-19 infection control should be notified immediately, and the local Centers for Disease Control and Prevention (CDC) should be notified as well. The receiver should bring the patient to the isolation room *via* the emergency lane under Level III protection. Furthermore, the patient will wait to be brought by the CDC personnel to the designated COVID-19 treatment facility for treatment. In the event of an emergency requiring immediate treatment or surgery for the four above categories of people or patients with unknown epidemiologic history, the patients should be placed in an isolation consultation or isolation surgery room for consultation or surgery under Level III protection (33), with proper isolation and disinfection measures and timely screening for SARS-CoV-2 infection after treatment (34, 35). If the vital signs are stable, the patient should be promptly transferred to a designated treatment facility for the subsequent consultation and treatment of COVID-19. The personnel involved in the practice need to be isolated at home or centrally for 14 days and must test negative for nucleic acid before returning to work.

However, the emergency passage can only be temporarily developed according to the current building structure, and there is no special emergency passage. When transferring the four categories of people, it is difficult to completely avoid crowds. It is only possible to arrange personnel to evacuate the crowd in time to ensure the transfer route's safety as far as possible when a similar situation occurs. Therefore, in the future, the buildings of dental hospitals or dental institutions should set up special emergency passages for the timely and safe evacuation of special groups of people in response to similar public health emergencies.

## System for Starting the Practice of Dental Institutions During a Pandemic

### Implementation of a Three-Level Pre-screening and Triage System for Dentistry

**Figure 2:**
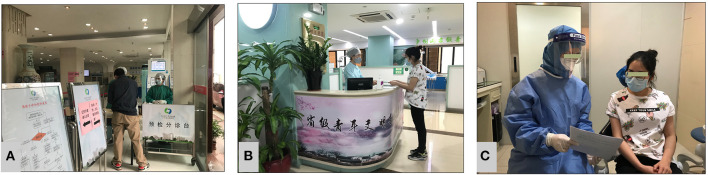
Three-level pre-screening and triage. **(A)** Level 1 lobby triage. **(B)** Level 2 clinic front desk triage. **(C)** Level 3 consultation space triage.

Guarding should be implemented at the four entrances of the dental institution: the entrance of the main gate of the dental hospital or dental clinic, the entrance of the outpatient lobby on the first floor, the entrance of the front desk of the clinic, and the entrance of the ward Three-level pre-screening and triage should also be implemented for the outpatient clinic: the first level at the outpatient lobby, the second level at the front desk of the clinic, and the third level at the dental chair (36). The staff at the outpatient hall is under Level II protection. The staff at the front desk of the clinic is under Level I protection. The staff at the dental chair is under Level II protection (see Table 1, Figure 2 for details). Different levels of protection for different positions should also be implemented, at least three epidemiological history inquiries and temperature measurements for all patients should also be conducted, and the nucleic acid test reports and CT reports should also be checked when necessary. Each level in the three-level pre-screening and triage is indispensable.

The pre-screening and triage site should be marked, relatively self-contained, well-ventilated, and equipped with rapid hand disinfectant, surface disinfectant, PPE, and temperature detection devices. The pre-screening and triage counters and items should be disinfected every 2 h and whenever contamination is encountered.

### Differentiation of Dental Practice Items by Risk Level

The high-, medium-, and low-risk areas released daily by the state are divided according to the zoning and classification criteria, which are refined to community-based units. The risk level of the pandemic is dynamically adjusted and announced on time. The pandemic is handled according to the principles of “early, small, strict, and practical” (37). The specific classification principles are (Table 2): ① High-risk areas are defined as areas with more than 50 new cases of COVID-19, with a cluster outbreak within the last 14 days; ② Medium-risk areas are defined as areas with new confirmed cases of COVID-19 within 14 days, but with the total number of confirmed cases of COVID-19 not exceeding 50 cases, or areas with the total number of confirmed cases of COVID-19 exceeding 50 cases, but with no cluster outbreak within 14 days; ③ Low-risk areas are defined as areas with no confirmed cases of COVID-19 or no new cases of COVID-19 for 14 days.

**Table 2:** Hierarchical differential guidelines for dental institutions to start practice.

	**High-risk areas**	**Medium-risk areas**	**Low-risk areas**
Consultation settings	Separate consultation rooms are open, and only one consultation space is available in large multi-place rooms	A separate consultation room starts a dental practice, with multiple consecutive consultations with one consultation space interval.	Resumption of daily consultation schedule
Dental practice items allowed	The entire hospital should be closed for routine outpatient care, and a 24-h emergency clinic should be set up, with a focus on emergencies and the use of low-speed turbines if necessary	Suspend high spattering operations; remaining items can be phased in, using low-speed turbine handpieces if necessary	Resumption of daily starting dental practice program
Patient sources	Emergency	A small number of online or telephone appointment numbers and emergency numbers in different time slots	The appropriate number of online or telephone appointments and emergency numbers in different time slots
Patient visit requirements	Need to have nucleic acid test report within 7 days and CT report if necessary	Present nucleic acid test report and CT report within 7 days if necessary	Nucleic acid test report within 7 days for high spattering operations and CT report if necessary
No anomalies in health code green code or communication big data	Need	Need	Need
Disinfection of objects surfaces	Terminal disinfection four times a day, disinfection of the dental chair after each consultation, and terminal disinfection of dental chairs after splattering operations	Terminal disinfection four times a day, disinfection of the dental chair after each consultation, and terminal disinfection of dental chairs after each splattering operation	Twice-daily Terminal disinfection, disinfection of the dental chair after each consultation, and terminal disinfection of dental chairs after each splattering operation
Floor and wall disinfection	Avoid dry sweeping, disinfect four times a day, disinfect wall height 2–2.5 m, disinfect anytime there is contamination	Avoid dry sweeping, disinfect twice a day, disinfect walls at the height of 2–2.5 m, disinfect whenever there is contamination	Avoid dry sweeping, disinfect twice a day, disinfect walls at the height of 2–2.5 m, disinfect whenever there is contamination
Surface disinfectants	1,000 mg/L chlorinated disinfectant solution can be used; see “8.1 Surface disinfectants” for other details	A disinfectant solution containing 500–1,000 mg/L chlorine can be used; see “8.1 Surface disinfectants” for other details	500 mg/L chlorinated disinfectant can be used; see “8.1 Surface disinfectants” for other details
Elevators	Elevator keys and cabs are disinfected every 2 h, and whenever there is contamination	Elevator keys and cabs are disinfected every 2 h, and whenever there is contamination	Elevator keys and cabs are disinfected every 2 h, and whenever there is contamination
Elevator disinfectants	500 mg/L chlorinated disinfectant or 75% alcohol	Same as high-risk areas	Same as high-risk areas
Toilet	Disinfect four times a day and whenever there is contamination	Disinfect four times a day and whenever there is contamination	Disinfect twice daily and whenever there is contamination
Toilet disinfectants	1,000 mg/L chlorinated disinfectant	Same as high-risk areas	500 mg/L chlorinated disinfectant
Air	Use the air disinfector throughout the practice, and use the UV lamp for 30–60 min when no one is around, and open the windows twice a day for more than 30 min each time	Same as high-risk areas	Same as high-risk areas
Air disinfectants	See “8.2.1 Air disinfectants” for details	Same as high-risk areas	Same as high-risk areas

This shows that the classification of the pandemic's risk level in each region is dynamic and constantly changing. Dynamic adjustments should be made in practice items, and dental chair arrangements according to the national and regional pandemic changes and the different risk levels of COVID-19 in different regions. Studies have shown that the bacterial load is five times higher after simultaneous dental treatment in multiple chairs, while this difference is reduced to two times higher with single chairs (38). Therefore, it is important to rationalize practice items and chair settings during a pandemic to achieve four-handed dentistry to reduce infection and occupational exposure risk. Furthermore, dental chairs should be set up in well-ventilated consultation rooms with air sterilization equipment (3, 38). The measures for performing dental practice during periods of different risk levels are as follows:

### High-Risk Areas

In such areas, dental clinics are completely closed, while dental hospitals, depending on their scale, retain 4–6 medical staff and one guide per day for dental emergencies and on-site consultations. A dedicated infection control specialist is also established to supervise and guide medical staff in infection control and achieve standard prevention. Patients attending the clinic must hold a green code and communication big data without abnormality. Furthermore, they must present the results of a nucleic acid test taken within 7 days and CT reports within 7 days only if necessary. Medical practice should be conducted in a separate room with good ventilation and sterilization equipment, and only one dental chair can be used in a large multi-chair room. The practice items are mainly for dental emergencies, and slow handpieces can be used for emergencies. The orthodontics department can receive return visits and suspend spattering operations, but the inpatient department of maxillofacial surgery must postpone elective surgery and only accept patients with dental emergencies requiring inpatient treatment. Patients are admitted in a single room, and only one person is allowed to accompany each patient. Patients and accompanying persons need to undergo nucleic acid detection and CT screening before being admitted to the hospital, and they can only be admitted if there is no abnormality. During hospitalization, the patient and accompanying staff should be monitored for body temperature and respiratory symptoms every day. They should not go out without permission. Nucleic acid testing and CT screening must be repeated after going out.

### Medium-Risk Areas

At this time, some dental outpatient consultations can be gradually resumed, and the number of the medical staff at work can be increased appropriately according to the number of patients. Except for periodontal treatment, dental implants, and dental preparations, which are highly spattering procedures, the rest of the procedures can be resumed gradually. Dental practices should be carried out in separate consultation rooms. Furthermore, two consecutive dental chairs in a large multi-chair room cannot be used simultaneously. Seats at an interval of one chair should be used. Attending patients should present their green code and communication big data without abnormalities. Furthermore, a COVID-19 nucleic acid test report and/or CT report taken within the last 7 days should be presented if necessary. During the medium-risk period, principles for admitting patients to the ward remain the same as those during the high-risk period.

### Low-Risk Areas

Daily dental outpatient practice will resume at this time. For highly spattering operations such as tooth extraction (except for deciduous teeth), periodontal treatment, and dental implants, patients will be required to hold a green code and communication big data without abnormalities, as well as present a 7-day COVID-19 nucleic acid test report and, if necessary, a 7-day CT report. Conversely, other procedures do not require a nucleic acid test report. All chairs can be used normally (except dental isolation chairs). The ward may add elective procedures as appropriate, and single-room admission should be adopted as far as possible. For multi-bed wards, patients should be placed one bed apart, windows should be opened regularly for ventilation in the ward, and the patients' and accompanying persons' temperature should be monitored. Moreover, respiratory symptoms should be observed daily during hospitalization. Nucleic acid testing and CT screening are still required before the hospitalization of patients and accompanying persons.

## Development of a System for the Management of Patients and Accompanying Persons in Dental Facilities

### Investigating the Epidemiological History

During the pandemic, each patient and the accompanying person must fill out the COVID-19 Epidemiological Questionnaire (39). The health code and communication big data trip cards should be reviewed, and the hospital should keep the questionnaire. The questionnaire's content should be dynamically adapted to the epidemiological characteristics of COVID-19, taking into account the daily release of areas at high risk of the pandemic.

For example, at the beginning of the pandemic, Wuhan was the center of the outbreak, so the location for investigating the epidemiological history was Wuhan. Any visits to Wuhan or contact with people who visited Wuhan within 14 days were ruled out (40), body temperature were tested, and respiratory symptoms were ruled out. On April 26, 2020, the COVID-19 patients in Wuhan were cleared, so Wuhan was no longer included in the epidemiological survey questionnaire.

### Provide COVID-19 Nucleic Acid Report and/or CT Findings as Necessary

Rapid sequencing of the SARS-CoV-2 genome has led to the development of real-time multiplex reverse transcription-polymerase chain reaction (RT-PCR) assays as the gold standard for detecting viral RNA and identifying patients with COVID-19 and asymptomatic carriers (41). Furthermore, it is currently the main type of test used to determine whether a patient is infected with SARS-CoV-2 in China. Patients with mild COVID-19 can have no pneumonia symptoms; therefore, CT results alone cannot be used as a criterion to rule out COVID-19 infection. However, nucleic acid testing can be affected by the disease's course, specimen collection, testing procedure, or testing reagents, among other factors. The test should be performed at a medical institution qualified for nucleic acid testing to improve the testing's positive rate. The specimens should be collected by professionals and sent for testing as soon as possible (3). The government qualifies nucleic acid testing institutions in China, and nucleic acid testing institution search, APPs, has been launched, through which the names and locations of testing institutions can be checked at any time.

### Strengthen Patient Waiting Management in Dental Institutions

When patients visit dental institutions, they must all wear masks, and no accompanying persons can enter. In the case of the elderly, children, or patients with limited ability to act due to illness, one accompanying person may be allowed to enter and is required to wear a mask throughout. Patients should also be screened for green codes and communication big data to confirm lack of abnormalities before entering. A dedicated person should maintain order on the site, instructing patients to maintain a minimum distance of 1 m from each other (42), be seated one chair away from each other, maintain good cough etiquette, and avoid gathering of people.

## Increase Self-Protection of Medical Personnel in Dental Institutions

### Selecting the Right Personal Protective Equipment

Since dental consultations are performed close to the patient, the operator's facepiece, eye mask, face mask, and operating arm are the areas closest to the patient and are the most contaminated (43). Thus, protection requirements should be at least Level II; Level III should be achieved when receiving the above four categories of people. Disposable protective equipment should be discarded after use and replaced when there is significant contamination with blood or body fluids. It is also necessary to avoid the overuse of PPE. Overlapping protective clothing is prone to breakage that is not easy to detect. It is more likely to be soaked with sweat during work, reducing the protective performance. Scientific and reasonable use of protective equipment can effectively reduce the risk of infection (44). The eye shields worn during operation often fog up, resulting in unclear vision, and are prone to occupational exposure. The anti-fogging method for the endoscope lens in the operating room can be borrowed; that is, iodophor can be evenly applied to the inner side of the eyepiece. The ionic iodine becomes molecular iodine, which plays an oxidizing role and forms a protective film that can last for 4–5 h against fogging.

Before the outbreak, dental practices generally used Level I protection with protective face shields or eye shields for highly spattering operations. The outbreak was so rapid that the vast majority of dental practices had inadequate protection measures in place. There was a shortage of protective materials, resulting in inadequate protective equipment for medical staff. If disposable gowns are not available, they can be temporarily replaced by cloth surgical gowns, which can be changed once every 4 h and washed and disinfected centrally. This can also have a protective effect (Figure 2A). In the case of sufficient materials, it is strongly recommended to use disposable isolation gowns, surgical masks, or medical protective masks. However, neither isolation nor surgical clothing can effectively protect the neck of medical staff, posing a risk of infection. Moreover, the use of disposable protective clothing is seldom applied during diagnosis and treatment in the Stomatology Department due to the high cost and shortage of materials. Therefore, the disposable shawl cap was quickly developed. This can be used with isolation or surgical clothing to strengthen the protective measures of medical personnel (Figure 2C), and standard prevention could be achieved insofar as high-risk environments are involved.

### Strengthening Hand Hygiene

Many studies have shown the critical role of healthcare workers' (HCWs) hands in transmitting microorganisms in the healthcare setting and ultimately to patients (45). Handwashing can therefore slow the spread of the virus (46). Nonetheless, before the outbreak, hand hygiene compliance was poor among HCWs (47–49). Studies have shown that hand sanitizer dispensers and gloves are the most contaminated PPE (50). Therefore, it is very important to improve compliance to and accuracy of hand hygiene. WHO also issues an annual global call to action to health workers through its SAVE LIVES: Clean Your Hands campaign, held annually in the month of May. This campaign aims to educate health workers and patients on the importance of effective handwashing, which has become more urgent with the COVID-19 pandemic.

Use the seven-step handwashing technique and master the five moments requiring handwashing: two before and three after, two before: before contact with the patient and before aseptic operation; three after: after contact with the patient, after contact with the patient's surroundings and related objects, and after exposure to body fluids. When handwashing is not available, adequate rapid hand sanitizer should be deployed in the best configuration within the medical staff's reach, which will reduce unnecessary walking of medical staff and make it easier to improve hand hygiene implementation.

With the improvements in hand hygiene implementation rate, the frequency of handwashing and hand disinfection has greatly increased, consequently followed by an increase in the incidence of hand eczema (51–53). Several studies have reported that oral medical staff have a high incidence of hand eczema owing to different factors of moisture-related operations such as frequent handwashing, increased irritation from hand disinfectants, and the long-term use of rubber gloves (54–57). Because hand eczema involves macules, papules, vesicles, edema, scaling, hyperkeratosis, fissures, etc. (58), skin damage increases the susceptibility of acquiring contact infection. Moreover, wearing gloves can aggravate the symptoms of hand eczema; therefore, people with hand eczema who have been treated with the corresponding drug treatments should still daub emollients, temporarily rest after work, and reduce hand stimulation to avoid aggravation (59, 60).

### Proper Donning and Doffing of Protective Equipment Are More Important

Wearing proper PPE can help medical staff reduce the risk of infection, but incorrectly donning and doffing PPE can expose medical staff to hazardous conditions that make them highly susceptible to infection. Multiple publications during the outbreak reported proper PPE donning and doffing (61–64), with hand hygiene implementation throughout the process of proper PPE donning and doffing. Since gloves are the most heavily contaminated protective equipment, both the Glove-in-Glove Technique and Bird Beak Technique methods of glove removal can be used (65) to reduce the chance of hand contamination. Proper donning and doffing of PPE are necessary to reduce the chances of infection effectively.

## Strengthen Infection Control and Prevention in the Dental Clinic Environment

### Sterilization of Object Surfaces and the Environment in the Dental Clinic

Studies have shown that coronaviruses can survive from 2 h to 9 days on surfaces of various materials such as plastic, metal, or glass (66, 67). Furthermore, self-service printers, desks/keyboards, desktops, door handles, medical equipment, utilities, and walls and floors are the most contaminated areas in the clinic (50), especially those that are subject to high-frequency use, such as elevator buttons and bathrooms. Therefore, the dental clinic should be kept clean and tidy, desktop items should be kept in cabinets as much as possible, and object surfaces should be properly disinfected to reduce environmental contamination.

Object surface disinfectant: ① use 75% alcohol to wipe object surfaces two times and allow action for 3 min (spray disinfection is prohibited); ② use a chlorine-containing disinfectant at a concentration of 500–1,000 mg/L to wipe or soak for 30 min; ③ use quaternary ammonium salt disinfectant at a concentration of 1,000−2,000 mg/L to spray or wipe; ④ use 0.1–0.2% peroxyacetic acid or 3% hydrogen peroxide to spray or soak for disinfection for an action time of 30 min. In cases of obvious pollution, water-absorbent materials containing 10,000 mg/L chlorine disinfectant can be used to wrap the pollutants and can be cleaned for disinfection again (68).

### Measures to Reduce Aerosols in the Dental Clinic

#### Air Purification

(1) The fresh air system should be turned on in the dental clinic while ensuring the cleanliness of the fresh air system air intake and its surroundings; when there is no fresh air system, the doors or windows should be opened to enhance air circulation (69). An outbreak of COVID-19 was reported in an air-conditioned restaurant in Guangzhou, China, due to poor air circulation without a fresh air system and ventilation windows (70). Ventilating the clinic by opening windows to inject fresh outdoor air directly into the clinic is one of the quickest and most effective methods to reduce the amount of aerosols in the air inside the clinic (38).

(2) Air disinfection machines with human–machine co-presence may be used and kept running at all times during operation. These can also be used in an unoccupied environment with UV lamp irradiation for at least 30–60 min. If the temperature is lower than 20°C or higher than 40°C and indoor humidity is >60%, the irradiation time needs to be extended, while the power of the UV lamp should be tested regularly and the UV lamp should be cleaned 1–2 times a week. Every day after the end of the consultation, 0.2% peroxyacetic acid or 3% hydrogen peroxide can be sprayed using the aerosol spray method, with the dosage calculated as 20–30 ml/m^3^ (1 g/m^3^), and ventilation after 60 min of disinfection action. A total of 15% peroxyacetic acid can also be used for heating fumigation, with the dosage calculated as 7 ml/m^3^ and ventilation after 1–2 h of fumigation action (68).

#### Patients Should Properly Gargle Before Dental Consultation

Several studies have confirmed that preoperative patient gargling reduces room air bacteria caused by dental procedures. Furthermore, during the COVID-19 pandemic, pre-consultation gargling should be performed with 1.5–3% hydrogen peroxide or 0.5–1.5% povidone-iodine for at least 30 s (71–73). Simultaneously, the patient should be instructed to gently spit into the gargling cup, use a strong aspiration device to aspirate the liquid from the gargling cup, and to try not to use or reduce the mouthwash basin's use to reduce droplet and aerosol production. SARS-CoV-2 has also been shown to be insensitive to chlorhexidine and Hibitane, so gargling with these solutions is not recommended (3, 74). However, recent studies have shown that in SARS-CoV-2-positive subjects, gargling with 1% hydrogen peroxide does not reduce oral viral load (75). However, the sample size in this experiment was small, and only RT-PCR was used as the detection method; thus, the statistical power of the results remains questionable. The author hopes that more studies can be conducted to assess the relationship between the use of mouthwash and SARS-CoV-2 to identify the type, method, and concentration of mouthwash that can reduce or even inactivate SARS-CoV-2 instantly, so that it can be applied in oral diagnosis and treatment to reduce the risk of infection.

#### Use Four-Handed and Six-Handed Operation for Oral Treatment, Prevention, and Control

According to several literature reports, the use of four-handed and six-handed operations in oral diagnosis and treatment can effectively improve work efficiency, reduce the work intensity of oral medical staff, and concomitantly achieve better standard prevention (76–81). Therefore, during the pandemic period, four-handed operations are a necessity for oral diagnosis and treatment (82, 83). Six-handed operations, if available, are better, as they can improve work efficiency and effectiveness, shorten the working time, reduce the time of medical personnel's exposure to aerosol pollution, and reduce the risk of infection. However, some institutions believe that the implementation of four-handed and six-handed operations requires more human resources, and the operation cost will be greatly increased. Therefore, currently, many oral hospitals and dental clinics involved in daily oral treatment operations do not apply four-handed or six-handed operation. The author's unit has practiced four-handed operations for many years; during this pandemic, we realized the superiority of four-handed operation in the prevention and control of the pandemic in dental institutions, and we believe that the effective implementation of four-handed operations will increase the economic benefits of oral institutions and patient comfort.

#### Application of Powerful Aspiration Devices

To effectively reduce splatter contamination from dental operations, the literature emphasizes and recommends using strong aspiration devices. Studies have shown that the use of strong aspiration devices could reduce droplet splatter area by 93%. A strong aspiration group reduced aerosol particles by ~90% compared to a weak aspiration group (84); thus, they should be used in conjunction with strong and weak aspiration in clinical operations. An extra-dental vacuum aspirator (EOVA) can be used, if conditions permit, to reduce droplet and aerosol contamination. The use of EOVA has been mentioned in the literature as an effective method to reduce air pollution in dental clinics. It is recommended for the treatment of patients with infectious diseases (85–88). EOVA devices were heavily promoted during the COVID-19 pandemic, but these devices were relatively expensive and not easily accessible across the board.

#### Use Anti-suction Turbo Dental Handpieces for Oral Treatment

In recent years, more attention has been directed toward the pollution of the dental treatment table water system. The pollution sources are mainly municipal water pollution and sudden stoppage of turbo dental handpieces, which causes patients' saliva and blood to flow through the turbo dental handpieces and into the lumen of the dental table water system (89, 90). The pollutants, after the back suction, will be ejected again the next time they are used, which can easily cause nosocomial infections (91). Although a variety of disinfectants have been used for the disinfection of dental treatment table water system in recent years (92, 93), it is possible to fundamentally solve the problem by using anti-suction turbo dental handpieces to prevent regurgitation. However, this will also increase the cost of oral institutions to a certain extent, and popularization and promotion may require time. Nevertheless, the authors believe that in the current pandemic period, effective prevention and control of each link are the first issues that oral doctors should consider. It is reported that 0.5 vol% H_2_O_2_ added to the water spray of dental handpieces drastically reduced the possibility of coronavirus spread during aerosol-generating dental procedures (94). Although these results still require confirmation, it may be a promising method to deal with water pollution in the future.

#### Use of Rubber Dams in Dental Practice

Studies have shown that using rubber dams during operations reduced colony counts by 82.7% (lamp-side sampling) and 83.9% (chest-side sampling) (95). Most studies have formed a broad consensus that rubber dams effectively reduced splatter transmission by 33% and reduced surface bacterial contamination by 80–99% during aerosol generation (96). Therefore, the use of rubber dams, where possible, can be effective in reducing aerosol formation during clinical operations.

## Dental Panoramic Radiography and Cone Beam Computed Tomography are Recommended for Oral Radiography During the Pandemic

Intraoral radiography, dental panoramic radiography, and cone beam computed tomography (CBCT) are commonly used imaging techniques in the Department of Stomatology. As mentioned earlier, SARS-CoV-2 can be present in saliva and aerosols (97–99). Since intraoral radiography easily induces nausea and cough, there is a high risk of transmission. Concomitantly, since it is necessary to put the sensor into the patient's mouth, there is also a risk of cross-infection. Dental panoramic radiography and cone beam computed tomography are, therefore, recommended during the pandemic (100, 101). However, some dental clinics are less equipped and only have intraoral radiography. The sensors can be covered with a disposable plastic film. After the film is soaked with 1,000 mg/L chlorine-containing disinfectant for 30 min, it is discarded into a medical waste bag (102), and the sensor is disinfected with 75% alcohol.

Radiologists should also conduct secondary protection. Patients should use povidone-iodine or hydrogen peroxide gargle for at least 30 s before undergoing oral radiology examination. After each patient's examination, 75% alcohol or 500 mg/L chlorine-containing disinfectant should be used to disinfect the examination room and equipment (103, 104).

## Sterilization of Dental Chairs Before and After Practice

### Disinfection of Dental Chairs Before Starting Dental Practice

Routinely turn on the dental chair water line rinse for 2–5 min before starting practice to drain the residual fluid in the pipe from the previous day's consultation; this can reduce the bacterial content in the pipe by one third (Table 2). For dental chairs with a pipe disinfection function, the pipe should be disinfected before the consultation. Conversely, for dental chairs without pipe disinfection function, the water pipe can be flushed and disinfected before starting dental practice with 500 mg/L of chlorine-containing disinfection solution, 3% hydrogen peroxide, and 5% silver peroxide ion, and the air pipe of the dental chair should be flushed for 20–30 s (105, 106). Several studies have demonstrated that daily pre-consultation rinsing can flush out plankton deposited in the pipeline the night before and reduce the number of bacteria in the pipeline.

### Disinfection of Dental Chairs Should Be Carried Out After Each Spraying Operation

During patient visits, the stain-proof film can be used to avoid stains in the frequently touched areas. At the end of each consultation, remove the stain-proof film, disinfect the dental chair's surface, and flush the pipes. A chlorine solution at 500–1,000 mg/L can be used for terminal disinfection of the dental chair after each splattering operation. The higher the concentration of chlorine-containing disinfectant, the more effective the disinfection, but the more corrosive it is to the pipes; therefore, this is an emergency practice measure under the COVID-19 pandemic.

### Terminal Disinfection

During the pandemic period, terminal disinfection using 500–1,000 mg/L of chlorine disinfectant is performed 2–4 times a day. Disinfection of the dental chair pipe should also be performed simultaneously. For dental chairs without the pipeline disinfection function, the same disinfection method for the dental chair pipe before starting practice should be carried out. After rinsing, the power should be turned off and the pipe should be left for 30 min and rinsed with clean water. The pipe disinfectant's concentration should then be tested to prevent the disinfectant from corroding the dental chair pipe.

## Dental Appliance Handling

According to the Regulation for Disinfection and Sterilization Technique of Dental Instruments (WS 506–2016), the hazard degree of oral instruments can be divided into three groups: critical dental instruments, semi-critical dental instruments, and non-critical dental instruments. According to these requirements, it is important to choose the appropriate disinfection and storage methods (107). Among them, semi-critical and non-critical oral instruments only need to be cleaned and preserved after disinfection. However, during the pandemic period, oral instruments should be sterilized using high temperature and high-pressure steam and stored separately (except instruments with low temperature resistance) (108). Only one person should conduct sterilization or disinfection to avoid cross-infection. Separating semi-critical and non-critical dental instruments after packaging sterilization greatly increases the operating costs of oral medical institutions, especially of smaller dental clinics. This will cause them to not sterilize their equipment, and the equipment will be sent to other oral medical institutions for sterilization. Such scenarios not only increase the operation cost but also increase the workload. However, only by doing an adequate job of infection control in every detail of oral diagnosis and treatment operation can we avoid cross-infection and protect the safety of doctors, nurses, and patients.

After use, dental instruments are placed separately and recycled promptly, depending on the instrument material, function, and disposal method (107). A disinfection-cleaning-disinfection process can be used to treat dental instruments during an outbreak. It is advisable to pre-treat dental instruments at the chairside, decontaminated by wiping with 75% alcohol. It has been shown that the contamination rate of instruments decreases substantially after chairside pre-cleaning, while the cleaning time can be shortened, and the damage rate of instruments can be reduced (109). After chairside pre-treatment, instruments can be placed in 75% alcohol for 30–60 min, or in a chlorine-containing disinfectant solution at 1,000–2,000 mg/L for 30 min, before being soaked in a 1:150 multi-enzyme solution and sealed. Instruments that cannot be soaked (e.g., high-speed turbine handpieces or scaling machine handles) are stored dry and sealed after surface pre-treatment and promptly recycled to the supply room for centralized cleaning and disinfection. Dental instruments used by the four categories of people should be sealed after immediate chairside disinfection and stored wet or dry in a special sealed box, recovered by the supply room specialist, and sent to the supply room for disinfection *via* a special channel. The supply room should make contingency plans for instruments after use by the four categories of people before starting a dental practice. Designated personnel should be appointed to clean and disinfect them, which should reach Level 3 protection, and separate cleaning rooms should be set up. Disinfection equipment should be marked with clear and conspicuous labels to distinguish them from other instruments to avoid cross-infection.

## Medical Waste Management for Dental Practice

Medical waste, a hazardous by-product, carries the risk of infection. During outbreaks of major infectious diseases, improper collection, storage, transportation, or disposal of medical waste will cause ecological pollution and increase the risk of disease transmission (110).

The dress code for cleaning personnel performing waste collection during an outbreak includes long-sleeved overalls, disposable hat, disposable surgical mask, disposable isolation gown, waterproof latex gloves, waterproof boots, and face shield. Recovery of items after use for the four categories of people should be under Level III protection.

A storage room for isolation items should be designated to recover items after use for the above four categories of people, and the special bags and sharps boxes for medical waste should be carefully checked to ensure that they are not damaged or leaking. Double-layered bags should be used for medical waste, with a gooseneck knot closure, to seal them in layers. Each bag and sharps box should be tied or affixed with a Chinese label, and the label content should include medical waste generation unit, generation department, generation date, and category. The items should be marked as having been used for the four categories of people to enhance the warning effect. Before leaving the contaminated area, these items should be disinfected by spraying the bag's surface with 1,000 mg/L chlorine disinfectant (pay attention to spraying evenly) or adding a medical layer waste bag on the outside prior to transportation to the government-designated COVID-19 patient medical waste disposal facility. At the end of the transport, the transportation tool should be disinfected with 1,000 mg/L chlorine-containing disinfectant solution (111).

## COVID-19 Vaccination

China has been developing and producing the COVID-19 vaccine and has gradually started vaccinating key populations (e.g., medical personnel, frozen food industry, and logistics) with the COVID-19 vaccine since the end of December 2020, with rollout gradually being extended to all citizens. As of 24:00, February 9, 2021, 40.52 million doses of vaccination have been administered nationwide. The authors received COVID-19 vaccination on February 4 and March 4, 2021, respectively, without adverse reactions. However, recently, a new study showed that SARS-CoV-2 had evolved into more than 800 different subtypes or branches since the outbreak at the end of 2019 (112), with some strains not even detectable by nucleic acid tests. Admittedly, the protection rate of the COVID-19 vaccine currently in use is difficult to determine. This is a major challenge for future pandemic prevention and control; therefore, pandemic prevention and control cannot be slackened.

## Limitations

These measures also have limitations. For example, a health code, a three-level pre-testing triage, three-level protection, and four-handed operation are easier to implement in countries with abundant medical resources. According to the literature, many countries are experiencing shortages of supplies during the pandemic. Because of poor detection capabilities and insufficient knowledge of infection prevention and control (IPC) protocols (113–118), it is difficult to implement these measures. But during the pandemic, we must strive to achieve standard prevention and to avoid cross-infection. The authors recommend the following measures in response to these situations.

- Set up an IPC team to develop an IPC policy and emergency plan, considering the available resources, to guide medical staff and patients to protect themselves during the pandemic. Strengthen IPC knowledge by training, including that in handwashing, PPE use, etc. (119).
- In case of insufficient medical staff, the number and frequency of patient visits should be reduced to decrease the pressure on medical staff. Also, reassign the medical staff posted in the pre-testing and triage positions when all oral diagnoses and treatments cannot be guaranteed with four-handed operations, which should be performed for as many high-spray procedures as possible, to reduce aerosol pollution.
- When there is no air disinfection equipment, the operation should be conducted in a well-ventilated consulting room.
- Manual instruments should be used as much as possible to reduce aerosol pollution (120). During periodontal treatment, manual scaling can be used instead of ultrasonic cleaning; use slow dental handpieces instead of high-speed handpieces when the use of handpieces is necessary.
- Frequent handwashing should be performed in situations where hand sanitizers are in short supply.
- When a mask is not obviously contaminated or damaged, it should be disinfected with vaporous hydrogen peroxide, ultraviolet germicidal irradiation, and moist heat (121, 122). However, this method is only to be used when masks are in short supply.
- Cloth isolation gowns or surgical gowns can be used instead of disposable gowns. Although either cloth or surgical gowns can be used, they exhibit higher bacterial detection rates than do disposable gowns (123); however, one should change the cloth or surgical gown every 4 h and have centralized cleaning and disinfection facilities available. These gowns can be used in an emergency in case of a shortage of disposable gowns, but they have a high permeability (124), and should be replaced when there is obvious contamination.
- In the case of shortage of disposable protective face shields, one shield can be used repeatedly, but it must be strictly disinfected and stored in a clean area. The commonly used disinfection methods are as follows: After wiping and disinfecting with 75% alcohol, irradiate under ultraviolet light for 1 h (125); soak in 1% sodium hypochlorite solution for at least 10 min; soak in 2% glutaraldehyde for 10 min, rinse with normal saline, and then air-dry; and ethylene oxide (ETO) sterilization (126). Without a disposable protective face shield, a simple protective face shield can be made using a transparent, flexible plastic film and rope (127).

## Conclusion

Although the global COVID-19 pandemic is still ongoing, dental care for patients with dental diseases cannot be stopped. We should learn from experience and actively implement pre-screening and triage to control the infection source, taking into account the pandemic trend and the above dynamic measures on dental practice under the pandemic. According to the etiological and epidemiological characteristics of the COVID-19 virus, and to its pathogenic and epidemiological characteristics, the key to infection control is the reasonable, correct, and effective use of disinfectants. Simultaneously, we should adequately implement infection control and prevention and cut off transmission routes. Additionally, we should achieve standard prevention, protect susceptible people, protect doctors and patients in both directions, and avoid cross-infection to ensure safe and smooth dental practice.

## Data Availability Statement

The original contributions presented in the study are included in the article/Supplementary Material, further inquiries can be directed to the corresponding author/s.

## Ethics Statement

Ethical review and approval was not required for the study on human participants in accordance with the local legislation and institutional requirements. Written informed consent for participation was not required for this study in accordance with the national legislation and the institutional requirements. Written informed consent was obtained from the individual(s), and minor(s)' legal guardian/next of kin, for the publication of any potentially identifiable images or data included in this article.

## Author Contributions

LL was responsible for the study concepts and design, and literature research. MZ was responsible for the clinical studies and experimental studies. XC was responsible for the data acquisition and analysis. SC was responsible for the statistical analysis. CX was responsible for the manuscript preparation. WX was responsible for the manuscript editing. LJ was responsible for the study design. XZ was responsible for the experimental studies. PC was responsible for the guarantor of integrity of the entire study, definition of intellectual content, and manuscript review. MR was responsible for the guarantor of integrity of the entire study and manuscript review. All authors read and approved the final manuscript.

## Conflict of Interest

The authors declare that the research was conducted in the absence of any commercial or financial relationships that could be construed as a potential conflict of interest.
