# Supplementary material for: Optimizing Safe Dental Practice During the COVID-19 Pandemic: Recommendations Based on a Guide Developed for Dental Practices in China
Source: Front Med (Lausanne). 2021 May 26;8:619357. doi: 10.3389/fmed.2021.619357 (PMC8187590; doi:10.3389/fmed.2021.619357)
Supplement: Supplementary file 1 [file Data_Sheet_1.docx]

Supplementary Material

# Supplementary Data

Southern Medical University Dental Hospital (Guangdong Provincial Dental Hospital)

Questionnaire on the epidemiological history of SARS-CoV-2 infection

Patient's name: ID (passport) number: Patient's temperature:

Name of accompanying person: ID (passport) number: Accompanying person's temperature:

Contact number:

1. Have you been to the following places in the past 14 days?

Hubei or Wuhan None

Other well-defined COVID-19 epidemic areas Specific countries

2. Have you had contact with the following people within 14 days?

Patients with fever or respiratory symptoms in Wuhan and its surrounding areas

Patients with fever or respiratory symptoms in communities with other reported cases in China

None

Patients with fever or respiratory symptoms returning from abroad

3. Have you been in contact with a person infected with SARS-CoV-2 (who tested positive for nucleic acid) within 14 days?

Yes, No

4. Have there been two or more cases of fever and or respiratory symptoms in a small area such as home, school, or workplace within 14 days?

Yes, No

5. Did the patient himself have any of the following discomforts during the 7 days?

Yes, Fever Cough Weakness Diarrhea/vomiting Other. No

6. Is there any assurance that the above is true?

Yes No (There is legal liability for concealment)

Signature of the patient:

Signature of the pre-screening and triage site:

Signature of the attending physician:

Date of visit:

| 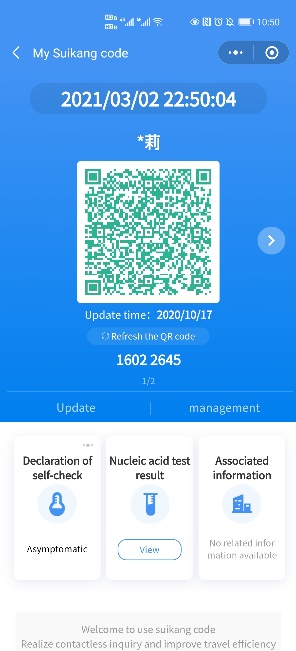 | 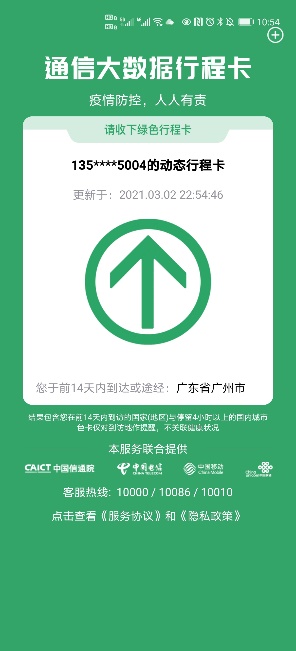 |
| --- | --- |
| **Health code** | **Communication big data trip cards** |
